# Novel polymorphisms in the prion protein gene (*PRNP*) and stability of the resultant prion protein in different horse breeds

**DOI:** 10.1186/s13567-023-01211-8

**Published:** 2023-10-17

**Authors:** Diego Sola, Rody Artigas, Diego R. Mediano, Pilar Zaragoza, Juan José Badiola, Inmaculada Martín-Burriel, Cristina Acín

**Affiliations:** 1 Centro de Encefalopatías Y Enfermedades Transmisibles Emergentes, Universidad de Zaragoza, IA2, IIS Aragón, 50013 Zaragoza, Spain; 2 Facultad de Veterinaria, Unidad Académica de Genética Y Mejora Animal, Universidad de La República, Ruta 8 Km18, 13000 Montevideo, Uruguay; 3 Laboratory of Biochemical Genetics (LAGENBIO), Faculty of Veterinary, Institute for Health Research Aragon (IIS Aragón), AgriFood Institute of Aragon (IA2), University of Zaragoza, Miguel Servet 177, 50013 Zaragoza, Spain; 4 Centro de Investigación Biomédica en Red de Enfermedades Neurodegenerativas (CIBERNED), Instituto Carlos III, 28029 Madrid, Spain

**Keywords:** Prion disease, prions, transmissible spongiform encephalopathy, *PRNP*, polymorphisms, horse

## Abstract

Prion diseases are fatal neurodegenerative disorders in which the main pathogenic event is the conversion of the cellular prion protein (PrP^C^) into an abnormal and misfolded isoform known as PrP^Sc^. Most prion diseases and their susceptibility and pathogenesis are mainly modulated by the *PRNP* gene that codes for PrP. Mutations and polymorphisms in the *PRNP* gene can alter PrP^C^ amino acid sequence, leading to a change in transmission efficiency depending on the place where it occurs. Horses are animals that are considered to be highly resistant to prions. Several studies have attempted to identify polymorphisms in the *PRNP* gene that explain the reason for this high resistance. In this study, we have analysed 207 horses from 20 different breeds, discovering 3 novel *PRNP* polymorphisms. By using computer programmes such as PolyPhen-2, PROVEAN, PANTHER, Meta-SNP and PredictSNP, we have predicted the possible impact that these new polymorphisms would have on the horse prion protein. In addition, we measured the propensity for amyloid aggregation using AMYCO and analysed the lack of hydrogen bridges that these changes would entail together with their electrostatic potentials using Swiss-PdbViewer software, showing that an increased amyloid propensity could be due to changes at the level of electrostatic potentials.

**Supplementary Information:**

The online version contains supplementary material available at 10.1186/s13567-023-01211-8.

## Introduction

Transmissible spongiform encephalopathies (TSE) or prion diseases are a group of neurodegenerative diseases, with fatal outcome, caused by a conformational change of the cellular prion protein (PrP^C^), being transformed into the pathogenic form PrP^Sc^. Prion diseases affect humans as well as domestic and wild animals. Sixteen different prion diseases have been described, seven in animals and nine in humans [1, 2]. In the case of animal diseases, the main ones are scrapie in sheep and goats, chronic wasting disease in cervids and the most relevant of all due to its zoonotic nature, bovine spongiform encephalopathy (BSE). BSE cases were also observed in wild ungulates, primates and felines due to consumption of feed containing ruminant meat and bone meal, beef or having been in close proximity to BSE-infected cattle [3–5]. Despite the wide variety of hosts, to date, prion diseases have not been detected in horses.

It has been recognized that polymorphisms in the PrP encoding gene (*PRNP*) are impacting susceptibility or resistance to prion diseases [6]. In humans, codons 129 and 219 are very important in terms of susceptibility to prion diseases [7–9]. G127V and E219K polymorphisms have also significant protective or modifying effects in acquired or sporadic diseases [2–10]. In sheep, more than 65 polymorphisms in PrP^C^ primary structure have been reported [11], although most appear to have no effect on scrapie susceptibility. However, polymorphisms at codons 136, 154 and 171 are determinant [12]. Of the possible variants, the genotypes 136V154R171Q/136V154R171Q, 136V154R171Q/136A154R171Q and 136A154R171Q/136A154R171Q have been associated with classical scrapie susceptibility, while the 136A154R171R/136A154R171R genotype has been associated with a higher level of resistance to classical scrapie [13–16]. On the other hand, susceptibility to atypical scrapie is linked to codon 141 [17, 18]. In the case of goats, various studies of the *PRNP* gene have shown 17 silent mutations and more than 40 amino acid substitutions, of which at least eight appear to be associated with scrapie resistance: G127S, I142M, H143R, N146S/N146D, R154H, R211Q, and Q222K [19–22]. Current data suggest that S146 and K222 confer each strong, if not complete, resistance to classical scrapie infection [23–26]. Other studies showed that the K222 polymorphism prolonged the incubation period of goats intracerebrally inoculated with bovine BSE, but did not have a strong effect against caprine BSE [27].

Several studies in deer have shown an association between polymorphisms in the *PRNP* gene and the modulation of chronic wasting disease (CWD). The first polymorphism described as protective of the disease was M132L and was discovered in Rocky Mountain elk (*Cervus elaphus nelsoni*) [28]. Recent studies suggest a lower susceptibility to CWD with the L132 polymorphism [29, 30]. Subsequently, mule deer (*Odocoileus hemionus*) homozygous for serine at codon 225 (S225) were found to have an increased susceptibility to CWD infection [31]. Other studies have described numerous polymorphisms that affect susceptibility to this disease. G65E, Q95H, G96S and A116G polymorphisms have been detected in white-tailed deer (*Odocoileus virginianus*) [32, 33], the latter three being associated with a lower susceptibility to CWD [33–35]. Other polymorphisms have been described in non-American cervid species, such as European elk (*Alces alces*) with K109Q polymorphism, reindeer (*Rangifer tarandus*) with N176D and S225Y polymorphisms and hyrax (*Dama dama*) with Q226E polymorphism [36]. In dogs, resistance to BSE and CWD has been attributed to the presence of two negatively charged amino acids, D163 and E163. This variant is characteristic of the canid family [37]. This misfolding resistance has recently been experimentally demonstrated in a mouse model expressing dog PrP^C^ [38].

There are few studies about the genetic variability of the *PRNP* gene in horses. Kim and Jeong detected a single nucleotide polymorphism (SNP), c.525C> A (N175K) at the *PRNP* coding region of 201 Thoroughbred horses [39]. More recently, by analysing the same gene region in Jeju and Halla horses, four mutations including a two synonymous SNP (c.−3A>G and c.570G>A) and one nonsynonymous SNPs (c.301T>A (W101R), have been detected [40]. So far, the polymorphisms of the equine *PRNP* gene have been only analysed in three horse breeds, Polymorphisms of this gene in other equine populations are still unknown.

The objective of this work was to analyse the variability of the *PRNP* gene coding region in horses from 20 different breeds, evaluating “in silico” the effect of the polymorphisms detected and the propensity to develop amyloid peptide aggregation.

## Materials and methods

### Animal samples

This study includes genomic DNA from 207 animals, including 20 breeds (Thoroughbred (*N* = 48), Pura raza española (*N* = 30), Burguete (*N* = 29), Jaca Navarra (*N* = 21), Standardbred (*N* = 18), Quarter Horse (*N* = 11), Nooitgedacht (*N* = 8), Westphalian (*N* = 8), Arabian (*N* = 7), Italian trotter (*N* = 5), Swedish Warmblood (*N* = 4), Selle Francais (*N* = 4), Oldenburg (*N* = 3), Hanoverian (*N* = 3), Haflinger (*N* = 2), Paint (*N* = 2), Appaloosa (*N* = 1), Percheron (*N* = 1), Wurttenberger (*N* = 1), Basuto pony (*N* = 1). These are unrelated animals from the DNA bank of the genetic laboratory of the University of Zaragoza, which belong to different distant regions and were obtained in different years from 2005 to 2019. These are animals from the DNA bank of LAGENBIO laboratory of the University of Zaragoza and from the ISAG (International Society for Animal Genetics) horse comparison test.

### Genetic analysis

The open reading frame (ORF) of *PRNP* gene (768 bp) was amplified from the genomic DNA with forward (PRNP-F: GGACACTGACACCCTCTTCATTTT) and reverse (PRNP-R: AAGGCCATCCTCATCCCACT) gene-specific primers. The PCR amplification was performed in a final volume of 50 μL, using the QIAGEN^®^ Taq PCR Core Kit, according to the manufacturer’s protocol. The reaction contained 20 pmol of each primer, 5 μL of 10 × PCR Buffer, 10 μL of 5 × Q-Solution Buffer, 1 μL of dNTP mix (10 mM), 2.5 U of Taq DNA polymerase and 5 µL of 80 ng/μL DNA. DNA amplification was performed using an S-1000 Thermal Cycler (Bio-Rad, Hercules, California, USA) under the following thermocycling program conditions: denaturation at 94 °C for 2 min, 35 amplification cycles of denaturation at 94 °C for 45 s, annealing at 60 °C for 45 s, and extension at 72 °C for 1 min 30 s; followed by a final 10 min extension at 72 °C. Amplicons were analysed by electrophoresis on a 1.0% agarose and purified using the vacuum manifold from Millipore® at 24 Hg of pressure for 3 min. PCR-amplified fragments on both strands were sequenced by thecompany Stab-Vida (Portugal) and the chromatograms were analysed using Chromas 2.6.6. (Technelysium Pty Ltd, Australia).

### Evaluation of the biological impact of nonsynonymous SNPs on horse PrP

The potential impact of nonsynonymous SNPs on horse PrP was evaluated by Polyphen-2 (Polymorphism Phenotyping v2), PANTHER 17.0, and PROVEAN web server (Protein Variation Effect Analyzer). Polyphen-2 is a program that predicts the possible impact of an amino acid substitution on the structure and function of a protein using straightforward physical and comparative considerations. As a result, three types of predictions can be obtained depending on the resulting score (0.0 to 1.0): “Benign”, “Possibly damaging”, and “Probably damaging” [41]. PANTHER estimates the probability of a particular nonsynonymous (amino-acid changing) coding SNP to cause a functional impact on the protein. It calculates the length of time (in millions of years -my-) a given amino acid has been preserved to the protein of interest. Depending on its “preservation time”, three results can be obtained: If the preservation time is above 450 my, the amino-acid changing could be “probably damaging”, if the preservation time is between 200 and 450 my, the amino-acid changing could be “possibly damaging”, and finally if the preservation time is below 200 my, the amino-acid changing could be “probably benign” [42]. PROVEAN is a web tool that predicts whether an amino acid substitution or has an impact on the biological function of a protein. Two types of results can be obtained depending on the resulting score: If the score is below −2.5 the variation effect is considered “neutral”, however, if the score is above −2.5, the variation effect is considered “deleterious” [43]. To evaluate the results, PredictSNP, a consensus software that assigns a combined score taking into account the scores obtained in other programmes such as MAPP, nsSNPAnalyzer, PANTHER, PolyPhen, PhD-SNP, SIFT, SNAP, was used [44]. Another consensus software used is Meta-SNP which integrates four existing methods: PANTHER, PhD-SNP, SIFT and SNAP [45].

### Assessment of amyloid propensity

To assess the amyloid propensity of horse PrP depending on the genetic polymorphisms, the AMYCO software was used. It utilizes an algorithm to predict amyloid fibril propensity from amino acid sequences [46].

### Statistical analysis

Genotype, allele, haplotype frequencies, Hardy–Weinberg equilibrium (breeds *n* > 10), and linkage disequilibrium (D′ and r^2^) according Lewontin [47] were calculated using the open software SHEsis [48]. D′ is the normalisation of the disequilibrium coefficient (D), its value ranges from −1 to 1, the higher the absolute value of D′, the greater the strength of linkage between loci. On the other hand, r^2^ is squared correlation between allelic values at two loci, the higher the value of r^2^ the greater the strength of linkage [47]. The frequency of Haplotypes 1 (Ht1) and 2 (Ht2) was measured by X^2^ test using GraphPad Prism version 8.0 (GraphPad Software, La Jolla, CA, USA).

### Haplotype network and 3D modeling of horse PrP

The haplotype network was built using the NETWORK 4.5.1 software using the “Median-Joining” algorithm [49], which relates in parsimoniously forms a data set on a single network.

Analysis of the effect of non-synonymous SNPs on prion structure was performed using Swiss-PdbViewer V4.1 software. Models for K175N and V182I amino acid changes were generated using the 3D model of horse PrP^C^ obtained from the Protein Data Bank. The prediction of hydrogen bonds was made according to Kim and collaborators [50]. Those predicted hydrogen bonds in the range of 1.2–2.76 Å from a “compatible” donor atom were considered. Using the surface charges of the exposed amino acids of the protein, the electrostatic potential was calculated using the Poisson-Boltzmann equation [51].

## Results

Depending on the species, the *PRNP* gene consists of 1, 2 or 3 exons. 1 for non-human primates, rabbits and birds, 2 for humans and horses and 3 for mice, sheep and cattle. The open reading frame (ORF) is contained entirely in the last exon. To investigate the genetic variation in the coding sequence of the horse *PRNP* gene, we performed a PCR amplification of exon 2 (836pb), which contains the full-length ORF, in a total of 207 horses from 20 different breeds. Direct sequencing of the amplicons revealed seven SNPs. Except for four previously reported polymorphisms (−3A>G, 301T>A (W101R), 525C>A (N175K) and 570G > A), we detected three novel variations: one synonymous SNPs (237T>C) and two nonsynonymous [5T>G (V2G), 544G>A (V182I)] (Additional file 1).

None of the breeds analysed were polymorphic for all markers at the same time. Regarding the SNP −3A>G, this was monomorphic for the A allele in all breeds except for Thoroughbred, Pura Raza Española, Jaca Navarra and Italian Trotter, where the A allele was the majority allele with a frequency ≥ 0.90 (Table 1). The SNPs c.5T>G (V2G) and c.544G>A (V182I) were monomorphic for the T and G allele respectively in all breeds, with the exception of Thoroughbred [c.5T > G (V2G)] where the T allele had a frequency of 0.98 and Jaca Navarra [c.544G > A (V182I)] where the G allele has a frequency of 0.98 (Table 1).

**Table 1:** **Genotypic and allelic frequencies for polymorphic sites of the horse *PRNP* gene**. Hardy–Weinberg Equilibrium (HWE). A: Alanine, G: Glycine, T: Threonine, C: Cysteine.

Polymorphisms/Breed	Genotype frequencies			Allele Frequencies		HWE (*P* value)
**c.−3A>G**	**AA**	**AG**	**GG**	**A**	**G**	
Thoroughbred (*N* = 48)	0.98	0	0.02	0.98	0.02	< 0.00001
Pura raza española (*N* = 30)	0.97	0.03	0	0.98	0.02	0.98
Jaca Navarra (*N* = 21)	0.95	0.05	0	0.98	0.02	0.98
Italian Trotter (*N* = 5)	0.80	0.20	0	0.90	0.10	–
**c.5T>G**	**TT**	**TG**	**GG**	**T**	**G**	
Thoroughbred (*N* = 48)	0.96	0.04	0	0.98	0.02	0.98
**c.237T>C**	**TT**	**TC**	**CC**	**T**	**C**	
Burguete (*N* = 19)	0.80	0.20	0	0.90	0.10	0.80
Jaca Navarra (*N* = 21)	0.90	0.10	0	0.96	0.04	0.94
**c.301T>A**	**TT**	**TA**	**AA**	**T**	**A**	
Thoroughbred (*N* = 48)	0.98	0	0.02	0.98		< 0.00001
Italian Trotter (*N* = 5)	0.80	0	0.20	0.80	0.20	–
**c.544G>A**	**GG**	**GA**	**AA**	**G**	**A**	
Jaca navarra (*N* = 21)	0.96	0.04	0	0.98	0.02	0.98

The novel c.237T>C polymorphism was only polymorphic in Burguete and Jaca Navarra, while c.301T>A (W101R) was only polymorphic in Thoroughbred and Italian Trotter, the rest of the animals being TT homozygous for both markers. In the breeds where these SNPs were polymorphic, the T allele stands out with a frequency ≥ 0.80 (Table 1).

The c.570G>A marker was monomorphic for the G allele in Thoroughbred, Westphalian and Nooitgedacht and was the allele with a frequency above 0.70 in all other breeds (Table 2). The c.525C>A (N175K) SNP was polymorphic in all breeds tested except Oldenburg, Hafliger and Paint (Table 2). In most breeds where c.525C>A (N175K) was polymorphic, the A allele was observed in higher frequency, except in Thoroughbred, Pura Raza Española and Westphalian, where the C allele was observed with a frequency of 0.68, 0.83 and 062 respectively (Table 2). All polymorphisms studied were found in Hardy–Weinberg equilibrium, except c.−3A>G and c.301T>A (W101R).

**Table 2:** **Genotypic and allelic frequencies for polymorphisms c.525C>A and c.570G>A of the horse *PRNP* gene for breeds with a number of N ≥ 5**. Hardy–Weinberg Equilibrium (HWE).

Breed	N	c.525C>A					HWE (*P* value)	c.570G>A					HWE (*P* value)
		CC	CA	AA	C	A		GG	GA	AA	G	A	
Thoroughbred	48	0.52	0.29	0.19	0.66	0.34	0.06	1	0	0	1	0	1
Pura raza Española	30	0. 67	0.33	0	0.83	0.17	0.55	0.93	0.07	0	0.97	0.03	0.96
Burguete	29	0.07	0.17	0.76	0.15	0.85	0.20	0.97	0.03	0	0.98	0.02	0.99
Jaca Navarra	21	0	0.23	0.76	0.12	0.88	0.80	0.67	0.33	0	0.83	0.17	0.61
Standardbred	18	0.06	0.44	0.50	0.28	0.72	0.90	0.72	0.22	0	0.83	0.17	0.76
Quarter Horse	11	0.09	0.54	0.36	0.36	0.64	0.84	0.91	0.09	0	0.95	0.05	0.96
Westephalian	8	0.50	0.25	0.25	0.62	0.38	–	1	0	0	1	0	–
Nooitgedacht	8	0.12	0.25	0.63	0.25	0.75	–	1	0	0	1	0	–
Arabian	7	0.14	0.42	0.43	0.36	0.64	–	0.70	0.30	0	0.86	0.14	–
Italian Trotter	5	0	0.40	0.60	0.20	0.80	–	0.40	0.60	0	0.70	0.30	–

**Figure 1:**
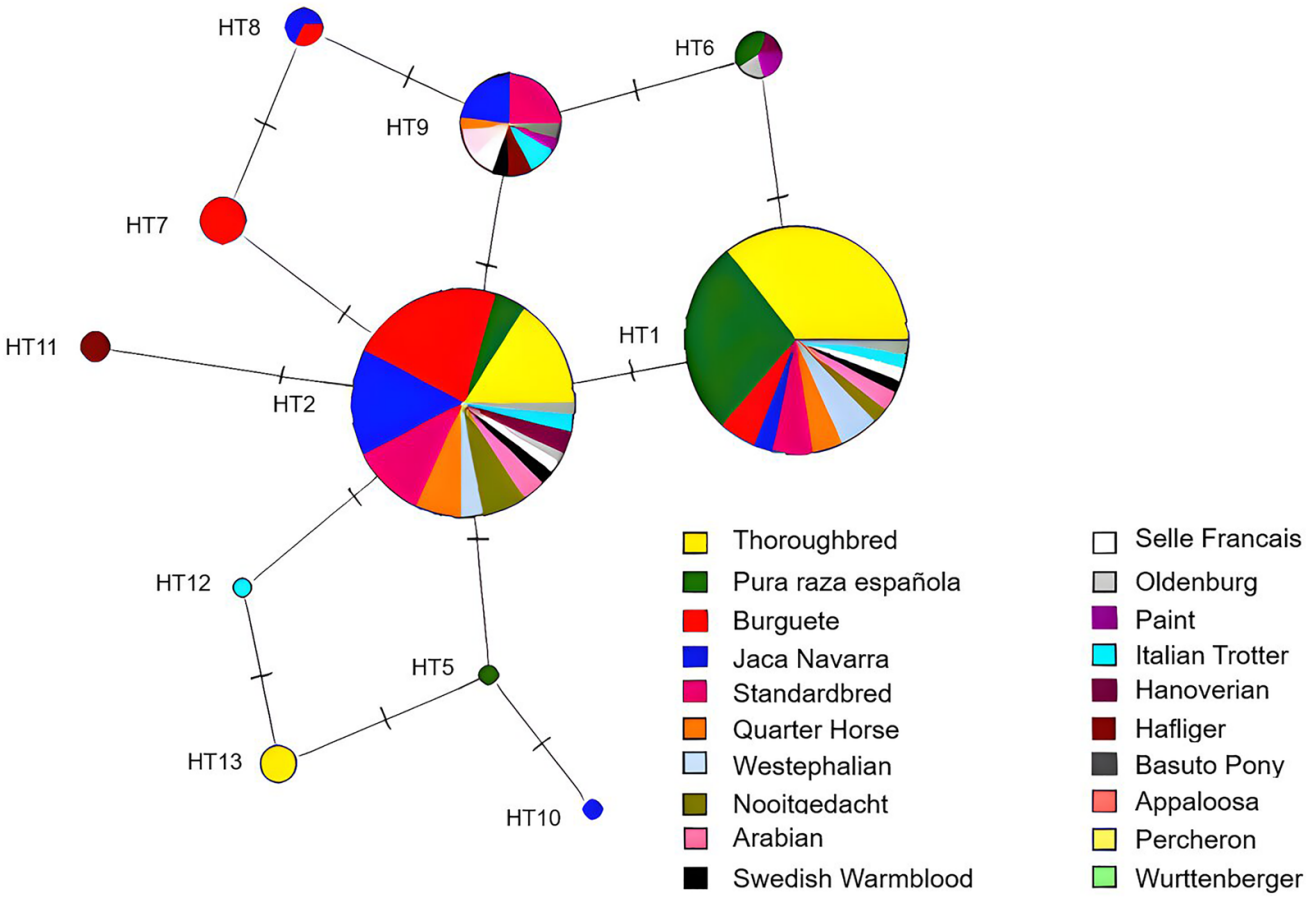
**Haplotype network for the horse *PRNP* gene**. The size of the circles is proportional to the frequency of the haplotype. The segments that cut the connectors indicate the number of mutational steps between each haplotype.

Thirteen different haplotypes (Ht) were found in all the animals studied, the frequency of each of them is shown in Table 3. Haplotypes Ht1, Ht2 and Ht9 were the most abundant ones with a frequency of 47%, 42% and 5.5% respectively, however, Ht1 and Ht2 were the only ones found in most of the breeds (Table 3). Haplotypes Ht3-Ht8 and Ht10-Ht13 were observed with a frequency lower than 1%. Haplotype network analysis showed that Ht2 might be the oldest haplotype (Figure 1). Linkage disequilibrium (LD) between the 7 polymorphisms found in the equine *PRNP* gene was studied using D′ and r^2^ coefficients. A strong linkage disequilibrium (r^2^ = 0.44 and D′ = 0.74) was observed between the markers c.−3A > G and c.301T>A (W101R) (Additional file 2).

**Table 3:** **Haplotype frequencies of 7 polymorphisms in the horse** ***PRNP* gene**.

Haplotypes	c.−3A>G	c.5T>G (V2G)	c.237T>C	c.301T>A (W101R)	c.525C>A (N175K)	c.544G>A (V182I)	c.570G>A	Frequency
Ht1	A	T	T	T	C	G	G	0.42
Ht2	A	T	T	T	A	G	G	0.47
Ht3	G	T	T	A	A	G	G	0.005
Ht4	A	G	T	T	C	G	G	0.005
Ht5	G	T	T	T	A	G	G	0.002
Ht6	A	T	T	T	C	G	A	0.012
Ht7	A	T	C	T	A	G	G	0.012
Ht8	A	T	C	T	A	G	A	0.007
Ht9	A	T	T	T	A	G	A	0.055
Ht10	G	T	T	T	A	A	G	0.002
Ht11	A	T	T	T	A	G	G	0.005
Ht12	A	T	T	A	A	G	G	0.002
Ht13	G	T	T	A	C	G	A	0.002

We estimated the potential impact that these nonsynonymous SNPs could have on horse PrP with PolyPhen-2, PROVEAN, PANTHER, Meta-SNP and PredictSNP. PolyPhen-2 predicted V182I (c.544G>A) and V2G (c.5T>G) as “Benign”. PROVEAN estimated both as “Neutral”. PANTHER dictates as "Possibly damaging" the V182I (c.544G>A) polymorphism and as “Possibly benign” the V2G polymorphism (c.5T>G). When considering consensus prediction with Meta-SNP and PredictSNP, both showed a neutral effect for V2G and V182I (Table 4).

**Table 4:** **Impact assessment of the non-synonymous V2G and V182I polymorphisms using Polyphen-2, PROVEAN, PANTHER, Meta-SNP and PredictSNP**.

Polymorphism	Method	Score	Prediction
c.5T>G (V2G)	PolyPhen-2	0.033	Benign
	PROVEAN	2.034	Neutral
	PANTHER	97	Possibly benign
	Meta-SNP	0.034	Neutral
	PredictSNP	83(expected accuracy)	Neutral
c.544G>A (V182I)	PolyPhen-2	0.084	Benign
	PROVEAN	−0.174	Neutral
	PANTHER	220	Possibly damaging
	Meta-SNP	0.26	Neutral
	PredictSNP	60(expected accuracy)	Neutral

**Figure 2:**
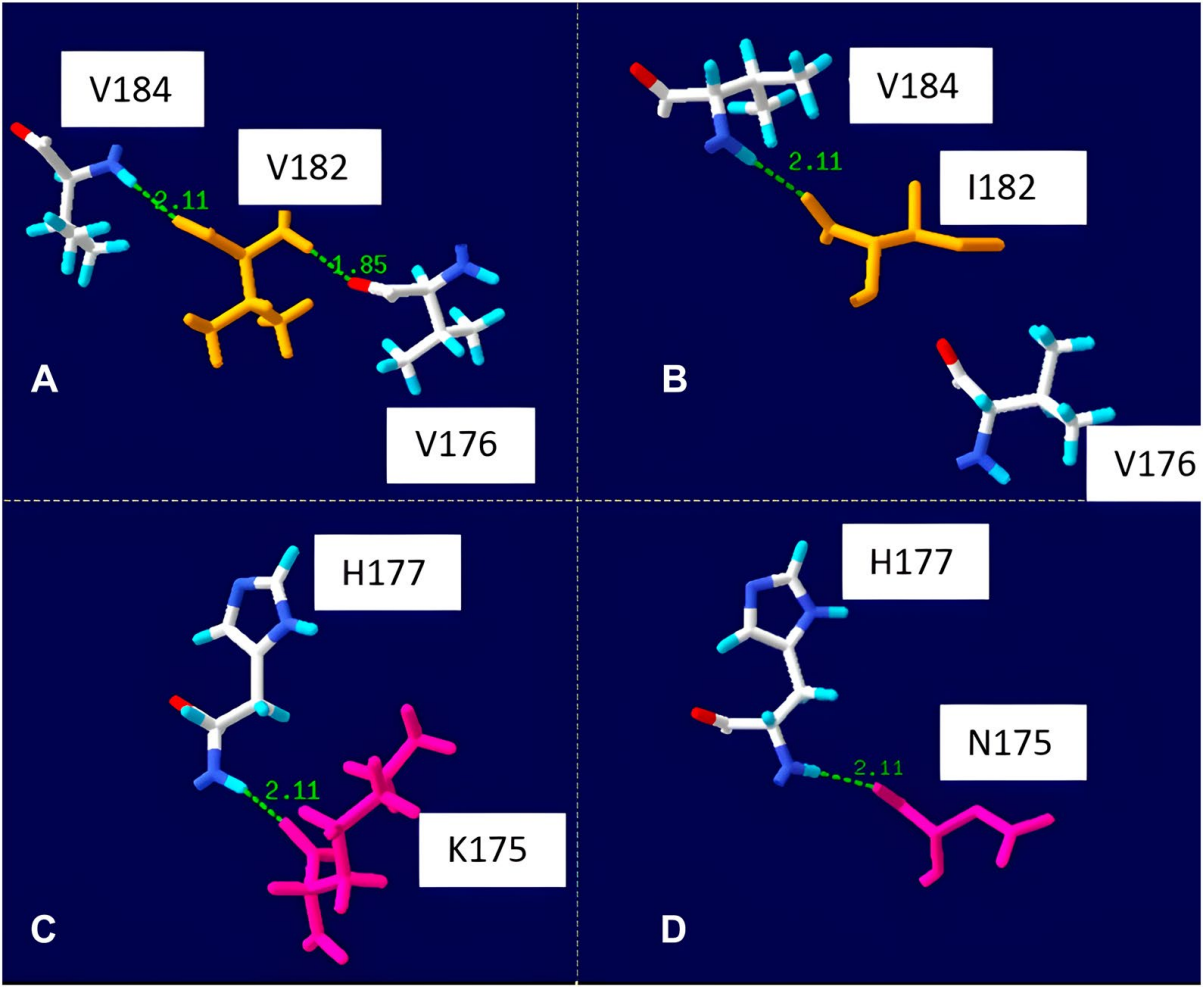
**Hydrogen bonds of horse prion protein (PrP)**. The dotted line and numbers in green indicate the hydrogen bonds and the distance (Å), respectively. **A** Effect of V182, **B** effect of I182 **C** effect of K175, **D** effect of N175.

The possible effect of non-synonymous changes on the structure of the horse PrP protein is shown in Figure 2. The Swiss-PdbViewer software predicted two hydrogen bonds for codon V182, one with V184 (2.11 Å) and one with V176 (1.85 Å). When the change to I182 occurs, the hydrogen bond with V176 is lost. In contrast to this, codon 175 maintains a hydrogen bond (2.11 Å) regardless of whether the amino acid present is a lysine (K175) or an asparagine (N175).

**Figure 3:**
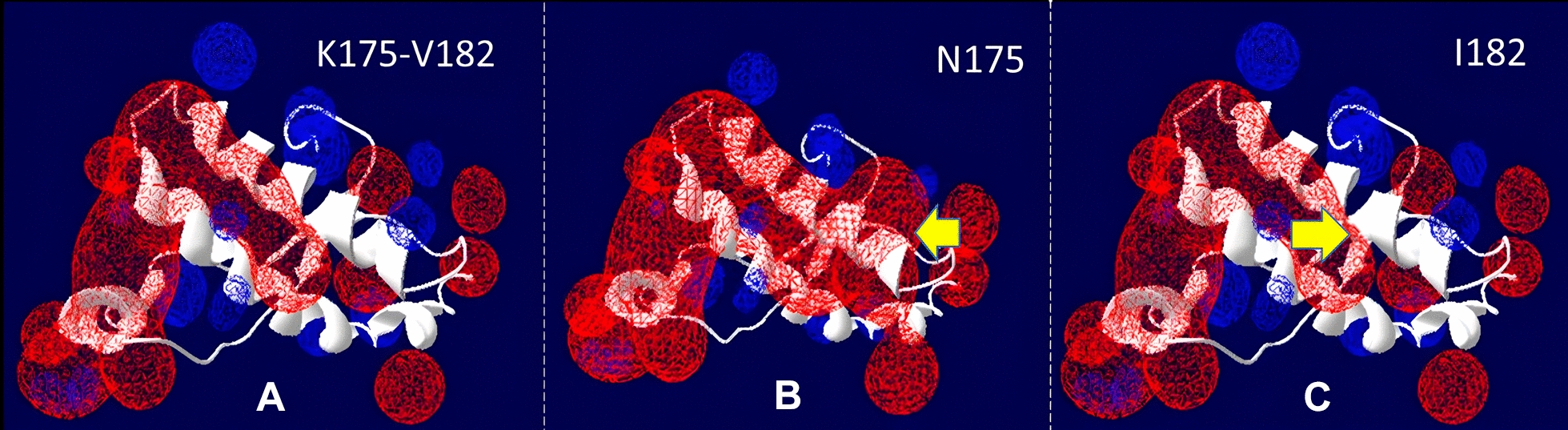
**Effect of amino acid substitutions N175K and V182I on the electrostatic potential of horse PrP**. The negative potential is shown in red and the positive potential in blue. Arrows indicate the site of substitution.

When evaluating the electrostatic potential, we observed changes depending on the different substitutions of amino acids in the protein (Figure 3). In the case of valine or isoleucine at codon 182, no electrostatic changes occur, however, in the presence of N175, the positive charge between codons 172–174 disappears, and the negative charge spreads across the region adjacent to the substitution, compared to the wild-type protein (K175).

Finally, horse PrP sequences were classified into 16 possible haplotypes according to the alleles of nonsynonymous SNPs (V2G (c.5T>G), W101R (c.301T>A), N175K (c.525C>A) and V182I [c.544G>A)] detected in this and previous work. The AMYCO software gives values between 0 and 1, and two thresholds are typically used: 0.45 and 0.78, where a score < 0.45 indicates a low propensity to aggregate and a score > 0.78 refers to a high propensity to aggregate. The maximum value of amyloid propensity was 0.39 and was observed in the haplotypes 2 V/101W/175N/182 V (Ht1 and Ht6), 2 V/101W/175N/182I, 2 V/101R/175N/182 V (Ht13), 2 V/101R/175N/182I, 2G/101W/175N/182I, 2G/101R/175N/182 V and 2G/101R/175N/182I followed with a value of 0.09 by 2 V/101R/175 K/182I and 2G/101R/175 K/182I (Table 5 and Additional file 3). The maximum value is determined by the presence of 175N.

**Table 5:** **Evaluation of the aggregation propensities of prion protein using AMYCO**.

Haplotype	Score	Haplotype in this study
2 V/101W/175N/182 V	0.39	Ht1 and Ht6
2 V/101W/175N/182I	0.39	–
2 V/101W/175 K/182 V	0	Ht2, Ht5, Ht7, Ht8, Ht9 and Ht11
2 V/101W/175 K/182I	0	Ht10
2 V/101R/175N/182 V	0.39	Ht13
2 V/101R/175N/182I	0.39	–
2 V/101R/175 K/182 V	0	Ht3 and Ht12
2 V/101R/175 K/182I	0.09	–
2G/101W/175N/182 V	0	Ht4
2G/101W/175N/182I	0.39	–
2G/101W/175 K/182 V	0	–
2G/101W/175 K/182I	0	–
2G/101R/175N/182 V	0.39	–
2G/101R/175N/182I	0.39	–
2G/101R/175 K/182 V	0	–
2G/101R/175 K/182I	0.09	–

## Discussion

To date, no cases of prion disease have been reported in horses. However, both PrP structural stability and *PRNP* genetic polymorphisms are still being studied because of the importance of understanding the resistance of specific prion protein sites. In this study, we assessed the polymorphisms in the ORF of the *PRNP* gene in different horse breeds. As previously detected, a total of 4 SNPs were found in the ORF of the horse *PRNP* gene (c.−3A>G, c.301T>A (W101R), c.525C>A (N175K), and c.570G>A) [39, 40]. In addition, we found three novel polymorphisms (c.5T>G (V2G), c.237T>C c.544G>A (V182I)), one of them synonymous (237T>C) and two non-synonymous (5T>G (V2G), 544G>A (V182I)). The larger number of breeds analysed favours the identification of new variants and therefore the genetic variability observed in the present work is higher than in previous studies.

The SNPs c.−3A>G, c.5T>G (V2G), c.237T>C, c.301T>A (W101R) and 544G>A (V182I) were polymorphic in six breeds, observing that the frequency of the most frequent allele was above 0.8. The remaining breeds were monomorphic for the most frequent allele. Similar allele frequencies for the polymorphisms c.−3A > G and c.301T>A (W101R) were observed for the Jeju and Halla breeds, while Thoroughbred was monomorphic for those SNPs [39, 40]. SNPs c.525C>A (N175K) and c.570G>A were polymorphic in most breeds, with allele frequencies similar to those reported in Thoroughbred, Jeju, and Halla breeds [39, 40].

All polymorphisms detected were found to be in Hardy–Weinberg equilibrium except c.−3A>G and c.301T>A (W101R). The lack of heterozygous animals for these polymorphisms may be due to the fact that these animals come from different populations and the less frequent allele appears only in one homozygous horse. The out-of-equilibrium polymorphisms were the two that showed the highest linkage disequilibrium (r^2^ = 0.44 and D′ = 0.74), particularly in the Thoroughbred breed that has been considered to have high levels of inbreeding [52].

Since the haplotypes were generated considering synonymous and non-synonymous polymorphisms, several haplotypes can encode the same amino acid sequence. Therefore, the 13 haplotypes found produce 6 different amino acid sequences. Using Network software, we calculated the number of mutational steps between each of the haplotypes. While the frequency of Ht2 is not significantly different from that of Ht1 (X^2^ = 0.44; *P* = 0.50), and while genetic recombination within the *PRNP* gene cannot be excluded, Ht2 would be the oldest, as it is the most frequently observed, it is found in the center of the network, and therefore it is the one that has the greatest relationship with the rest of the haplotypes. Ht1 was the most frequent in the Thoroughbred, Pura Raza Española and Westphalian breeds. Although in our work we found three new SNPs, Ht1 was also the most frequent in other works in the Thoroughbred [39] and Jeju and Halla breeds [40]. The high frequency of Ht1 may be due to inbreeding effects or the founder effect of the breeds.

In this study, we evaluated the biological impact of nonsynonymous SNPs on horse PrP using POLYPHEN-2, PROVEAN, and PANTHER as Kim and colleagues had previously done by assessing N175K (c.525C>A) as "benign" [40]. The V2G (c.5T>G) polymorphism was categorised as “benign”. This polymorphism is found in N-terminal endoplasmic reticulum (ER) signal peptide [39], so it is eliminated when the mature protein is formed [53]. In the case of the V182I (c.544G>A) polymorphism PANTHER dictates as “Possibly damaging” and it is located in the α-helix at residues 175–196. The latter is probably due to the fact that the amino acid Valine at codon 182 is highly conserved among the different species, so a change to Isoleucine is detected by the programme as a “Possibly damaging” change. Similar results were obtained in a recent study in the case of W101R (c.301T>A) [40]. For V182I all other programmes, including the PredictSNP and Meta-SNP consensus methods predict a neutral effect of that protein change.

To estimate the amyloid propensity that different combinations of nonsynonymous SNPs could cause, we used the in-silico program AMYCO. The horse prion protein has a value of 0.39 in AMYCO. In this study, it was observed that the maximum values of amyloid propensity (0.39) are marked by the amino acid N at position 175. However, this change does not produce alterations in relation to the number of hydrogen bonds in codon 175, so it would not in itself explain the instability of the protein. A different situation occurs with codon 182, where the change to isoleucine produces the loss of the hydrogen bond, consistent with a slight increase in amyloid propensity. This may be due to reduced protein stability, as salt bridges play a key role in stabilising the secondary and tertiary structural elements of the prion protein [54].

Previous studies have shown that horse PrP has a key region in the highly structured globular domain, composed of three α-helix zones and two small antiparallel β-sheets [39]. Two structures, called the β2-α2 loop and salt bridges, have been reported to confer remarkable stability to equine PrP structure, allowing it to withstand adverse conditions [55, 56]. In *Drosophila* in vitro studies, the S167 amino acid found in this region was found to be a key residue in the stability of equine PrP as it appears to introduce changes in the globular domain that result in decreased β-sheet content and increased conformational stability [57]. These results further support the view that equine PrP^c^ is resistant to prion spread. Some authors have described an alternative replicative phenomenon known as "non-adaptive prion amplification" (NAPA) [58]. Initially, it was observed in mice transgenic for equine PrP^C^ (TgEq) that were inoculated with an experimental sheep scrapie isolate called SSBP/1. In these experiments, only a small number of animals developed prion disease, demonstrating that equine PrP^C^ could be misfolded. However, the surprise came when it was discovered that, in a second propagation cycle, these prions could not be transmitted to animals of the same transgenic line (TgEq), but could be transmitted with high efficiency to ovine transgenic mice (the species of origin of the isolate used). In other words, these prions could spread, but they did not adapt to the new PrP^C^, but retained their ability to infect/transmit. Furthermore, the biochemical and strain properties of the inoculated prions remained intact after NAPA.

Horses are used in a multitude of tasks as labour, for competition, and for food. Most are raised on farms, but there are also wild herds, and in both cases, they may come into contact with other mammals that are susceptible to prion diseases. Therefore, the interest in understanding why certain mammals are more resistant to prion diseases is even more remarkable in the case of horses. The fact that many breeds exist and are distributed throughout the world also points to the importance of multi-breed analyses such as this one. It is therefore of great importance to continue research on prion diseases in those animals that are more resistant to prion diseases, such as horses.

In conclusion, the horse *PRNP* gene presents a low level of polymorphisms in the coding regions; moreover, none of these changes implies a greater susceptibility to amyloid propensity. The amino acidic change N175K (c.525C>A) seems to be the one that produces the greatest instability at the level of the structure. This instability could be caused by charges in electrostatic potentials. Further studies will be necessary to validate the effect of altered hydrogen bonding and electrostatic potential on prion aggregation in the equine species.

### Supplementary Information

**Additional file 1**. **Electropherogram of all SNPs found, including newly found SNPs (c.5T>G, c.237T>A, c.544G>A). **Four colours indicate individual bases of DNA sequence (blue: cytosine, red: thymine, black: guanine, green: adenine).

**Additional file 2**. **Linkage disequilibrium (LD) among the 7 polymorphisms found in horse *PRNP* gene. **The linkage disequilibrium value was investigated for the 7 *PRNP* SNPs found using Lewontin’s D′ and r^2^ values. All polymorphisms detected were found to be in Hardy-Weinberg equilibrium except c.−3A>G and c.301T>A. A strong linkage disequilibrium (r^2^ = 0.44 and D′= 0.74) was observed between the markers c.−3A>G and c.301T>A.

**Additional file 3**. **Graphical representation of the AMYCO score. A** Haplotypes 2V/101W/175N/182V (Ht1 and Ht6), 2V/101W/175N/182I, 2V/101R/175N/182V (Ht13), 2V/101R/175N/182I, 2G/101W/175N/182I, 2G/101R/175N/182V and 2G/101R/175N/182 showed the maximum value of 0.39. **B **Haplotypes 2V/101R/175K/182I and 2G/101R/175K/182I showed a value of 0.09. **C **Haplotypes Ht2, Ht3, Ht4, Ht5, Ht7, Ht8, Ht9, Ht10, Ht11, Ht12 showed a value of 0.

## Data Availability

All data generated or analysed during this study are included in this published article.
