# Supplementary figures and images for: Novel polymorphisms in the prion protein gene (PRNP) and stability of the resultant prion protein in different horse breeds
Source: Vet Res. 2023 Oct 17;54:94. doi: 10.1186/s13567-023-01211-8 (PMC10583458; doi:10.1186/s13567-023-01211-8)

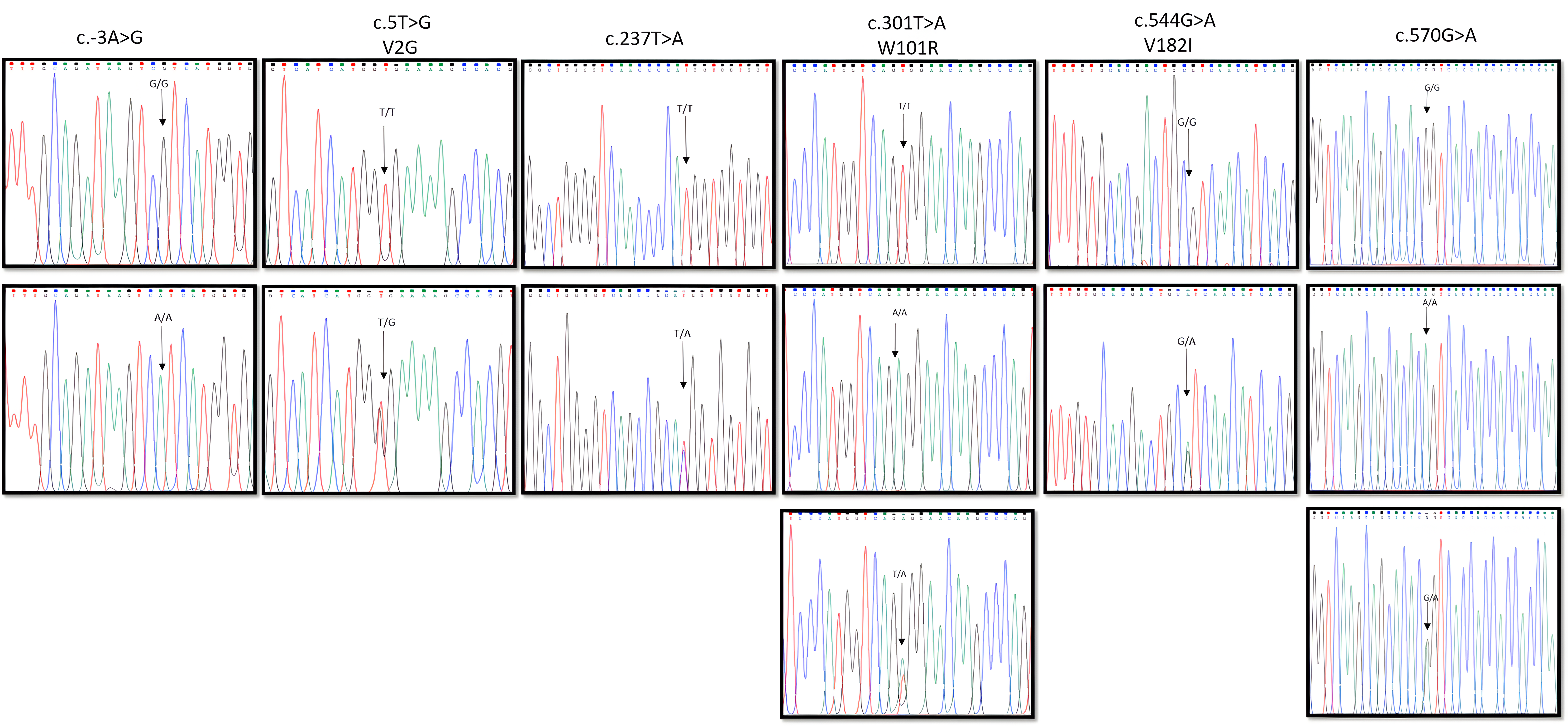

Supplement: Supplementary file 1 — Additional file 1. Electropherogram of all SNPs found, including newly found SNPs (c.5T>G, c.237T>A, c.544G>A). Four colours indicate individual bases of DNA sequence (blue: cytosine, red: thymine, black: guanine, green: adenine). [file 13567_2023_1211_MOESM1_ESM.png]

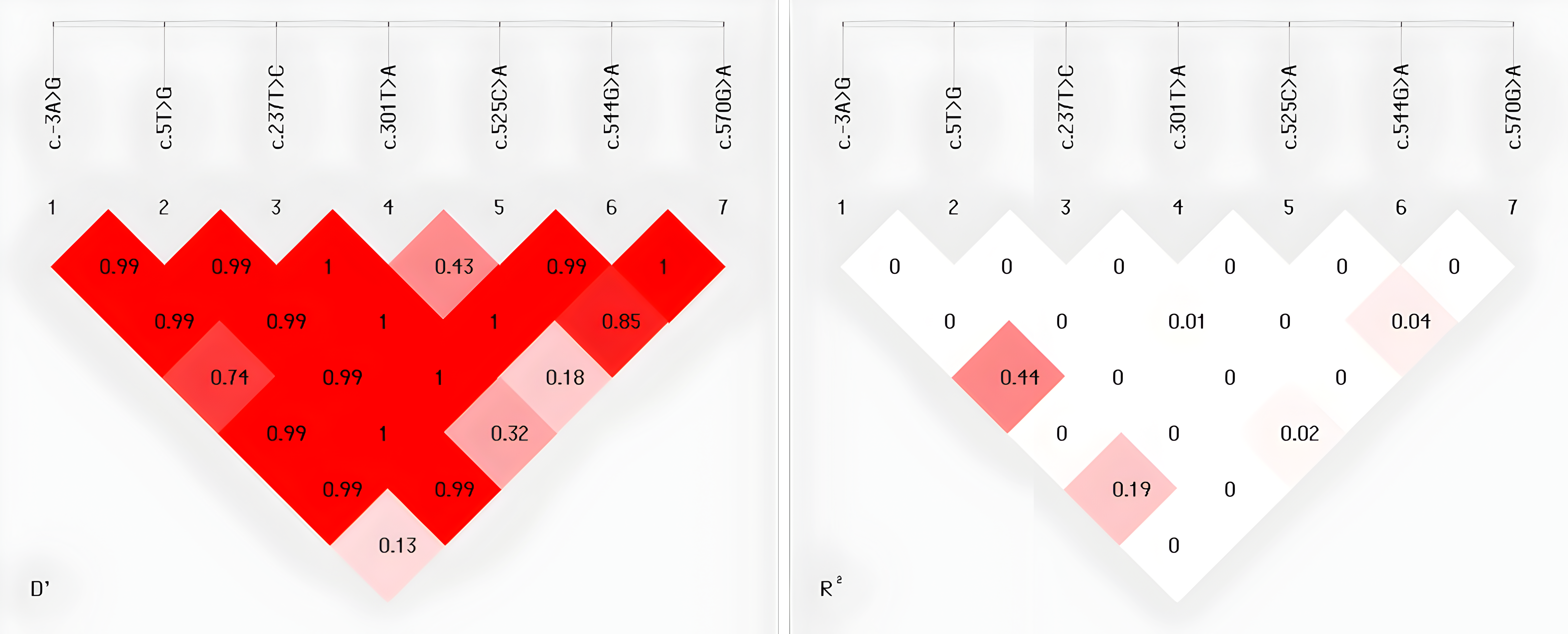

Supplement: Supplementary file 2 — Additional file 2. Linkage disequilibrium (LD) among the 7 polymorphisms found in horse PRNP gene. The linkage disequilibrium value was investigated for the 7 PRNP SNPs found using Lewontin’s D′ and r2 values. All polymorphisms detected were found to be in Hardy-Weinberg equilibrium except c.−3A>G and c.301T>A. A strong linkage disequilibrium (r2 = 0.44 and D′= 0.74) was observed between the markers c.−3A>G and c.301T>A. [file 13567_2023_1211_MOESM2_ESM.png]

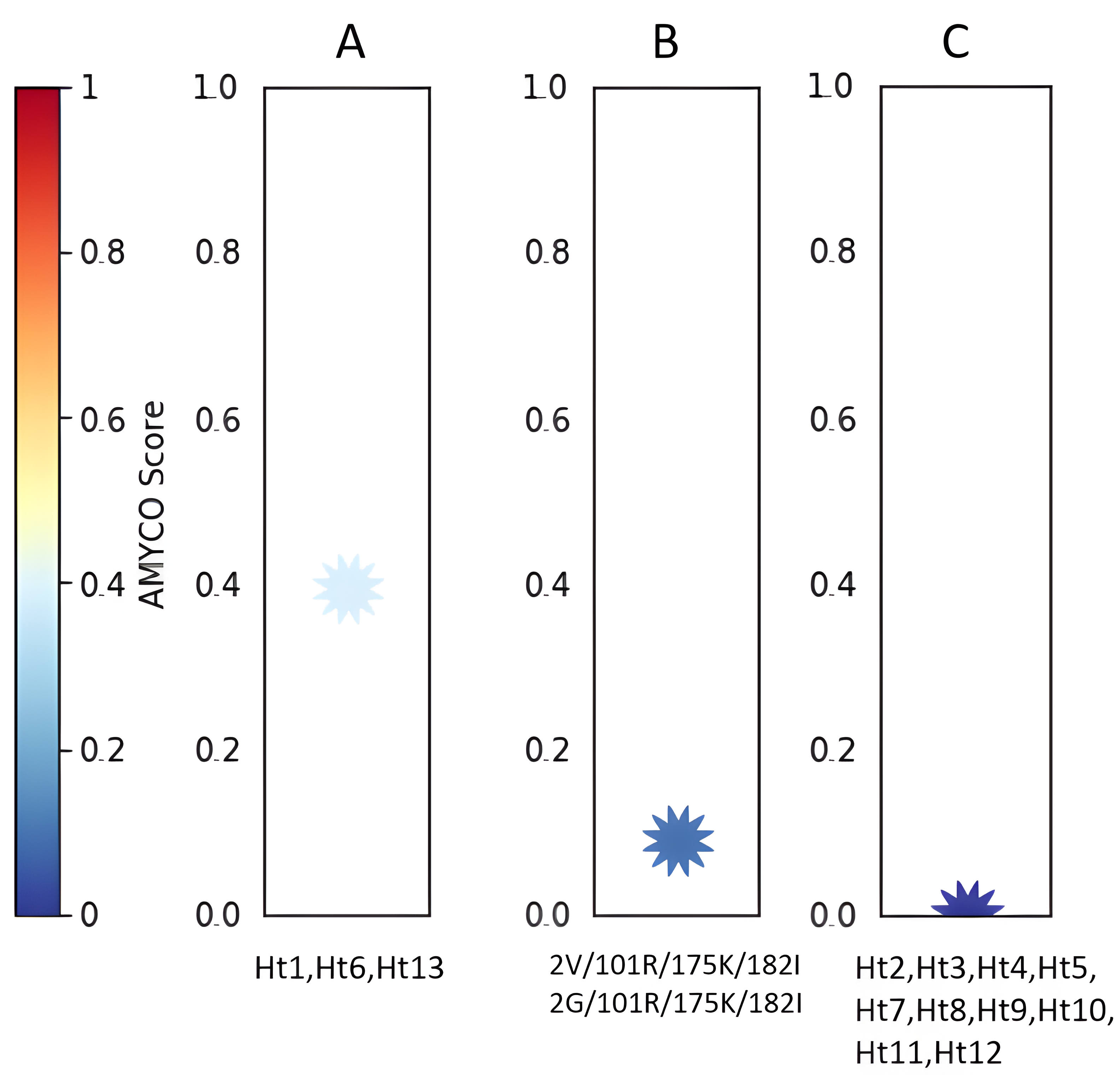

Supplement: Supplementary file 3 — Additional file 3. Graphical representation of the AMYCO score. A Haplotypes 2V/101W/175N/182V (Ht1 and Ht6), 2V/101W/175N/182I, 2V/101R/175N/182V (Ht13), 2V/101R/175N/182I, 2G/101W/175N/182I, 2G/101R/175N/182V and 2G/101R/175N/182 showed the maximum value of 0.39. B Haplotypes 2V/101R/175K/182I and 2G/101R/175K/182I showed a value of 0.09. C Haplotypes Ht2, Ht3, Ht4, Ht5, Ht7, Ht8, Ht9, Ht10, Ht11, Ht12 showed a value of 0. [file 13567_2023_1211_MOESM3_ESM.png]
